# Differential Effects of Painful and Non-Painful Stimulation on Tactile Processing in Fibromyalgia Syndrome and Subjects with Masochistic Behaviour

**DOI:** 10.1371/journal.pone.0015804

**Published:** 2010-12-28

**Authors:** Bettina Pollok, Vanessa Krause, Valery Legrain, Markus Ploner, Rainer Freynhagen, Ilka Melchior, Alfons Schnitzler

**Affiliations:** 1 Institute of Clinical Neuroscience and Medical Psychology, University of Dusseldorf, Dusseldorf, Germany; 2 Department of Neurology, University of Dusseldorf, Dusseldorf, Germany; 3 Department of Experimental-Clinical and Health Psychology, Ghent University, Ghent, Belgium; 4 Institute of Neuroscience, Université catholique de Louvain, Brussels, Belgium; 5 Department of Neurology, Technische Universität München, Munich, Germany; 6 Department of Anaesthesiology, Critical Care Medicine, Pain Therapy & Palliative Care, Pain Centre Lake Starnberg, Benedictus Krankenhaus Tutzing, Tutzing, Germany; 7 Department of Anaesthesiology, University of Dusseldorf, Dusseldorf, Germany; Tokyo Medical and Dental University, Japan

## Abstract

**Background:**

In healthy subjects repeated tactile stimulation in a conditioning test stimulation paradigm yields attenuation of primary (S1) and secondary (S2) somatosensory cortical activation, whereas a preceding painful stimulus results in facilitation.

**Methodology/Principal Findings:**

Since previous data suggest that cognitive processes might affect somatosensory processing in S1, the present study aims at investigating to what extent cortical reactivity is altered by the subjective estimation of pain. To this end, the effect of painful and tactile stimulation on processing of subsequently applied tactile stimuli was investigated in patients with fibromyalgia syndrome (FMS) and in subjects with masochistic behaviour (MB) by means of a 122-channel whole-head magnetoencephalography (MEG) system. Ten patients fulfilling the criteria for the diagnosis of FMS, 10 subjects with MB and 20 control subjects matched with respect to age, gender and handedness participated in the present study. Tactile or brief painful cutaneous laser stimuli were applied as conditioning stimulus (CS) followed by a tactile test stimulus (TS) 500 ms later. While in FMS patients significant attenuation following conditioning tactile stimulation was evident, no facilitation following painful stimulation was found. By contrast, in subjects with MB no attenuation but significant facilitation occurred. Attenuation as well as facilitation applied to cortical responses occurring at about 70 ms but not to early S1 or S2 responses. Additionally, in FMS patients the amount of attenuation was inversely correlated with catastrophizing tendency.

**Conclusion:**

The present results imply altered cortical reactivity of the primary somatosensory cortex in FMS patients and MB possibly reflecting differences of individual pain experience.

## Introduction

Touch and pain are intimately related modalities. Along this line, a modulating effect of painful stimuli on processing of tactile information has been evidenced in behavioural [1], [2], [3] and in neurophysiological [4], [5], [6], [7] studies. Using a conditioning test stimulation paradigm, Ploner et al. [7] demonstrated that preceding painful stimuli yield facilitation of subsequently applied non-painful tactile stimuli within S1 and S2 by means of MEG. Interestingly, facilitation was indicated by increased somatosensory evoked amplitudes of late S1 and S2 but not of early S1 responses. Conversely, a preceding tactile stimulus results in reduced early as well as late S1 amplitudes. These data suggest that the observed increase of the late S1 component might represent a neurophysiological correlate of the alerting function of pain. In order to shed further light on the functional significance of this modulating effect, the present study investigates patients with FMS and subjects with MB. While the latter valuate pain as positive and even pleasant under certain circumstances, for patients with FMS painful stimulation is highly aversive. Along this line, pain-related catastrophizing encompassing magnification and feelings of helplessness to the experience of pain has been noticed as a frequently occurring symptom in chronic pain states like FMS (reviewed in [8]). The present study aims at elucidating whether these two extreme ends of the spectrum of individual pain experience affect somatosensory processing.

FMS is a chronic non-inflammatory musculoskeletal pain condition characterized by diffuse widespread pain and increased sensitivity to pressure at characteristic tender points [9], [10]. Although the origin of FMS is largely unknown, it has been related to increased responsiveness of neurons known as facilitation of central nervous system pathways (reviewed in [10], [11], [12]). More precisely, imbalance between supraspinal inhibitory and excitatory modulation pathways has been related to the origin of chronic non-inflammatory muscle pain [13]. Along this line, reduced attenuation to non-painful somatosensory stimuli has been shown by electroencephalography (EEG) in FMS [14]. Additionally, evidence for increased responsiveness to painful stimuli in FMS has been found by means of behavioural [15], [16], [17] and functional magnetic resonance imaging (fMRI) studies [11] indicating deficits in endogenous pain inhibitory systems which normally protect against overstimulation [18], [19].

Masochistic behaviour is the tendency to derive sexual gratification from being physically or emotionally abused. Along this line, painful stimulation within a sexual context is frequently reported by MB subjects. Interestingly, the underlying mechanisms, particularly central mechanisms of pain perception have not been investigated so far. But, it seems to be reasonable that evaluating painful stimulation as positive might be related to alterations of central mechanisms of pain perception.

The present study aims at investigating to what extent the subjective evaluation of painful stimuli affects reactivity of somatosensory cortices by means of a conditioning test stimulation paradigm. We hypothesize differential effects on somatosensory excitability in subjects with masochistic behaviour and fibromyalgia patients.

## Materials and Methods

### Subjects and paradigm

All subjects gave their written informed consent prior to the study which was approved by the ethics committee of the University Hospital Duesseldorf and was in accordance with the declaration of Helsinki.

Ten fibromyalgia patients (54.0±2.9 years, mean ± s.e.m.; 9 female) participated in the present study. Eight of them were outpatients from the pain unit of the University Hospital Duesseldorf. Two patients were acquired through cooperation with the Institute of Neuropsychology and Clinical Psychology, Central Institute of Mental Health, Mannheim, Germany. All patients met the American College of Rheumatology (ACR) criteria for fibromyalgia [20]. The diagnosis was confirmed by a chronic pain expert (R.F.). Patients with additional diseases were excluded from the study. Mean duration of disease was 12±4.8 years. Clinical pain ratings prior to the MEG recordings were determined by means of a numerical rating scale ranging from 0 (no pain) to 10 (worst possible pain). Additionally, tender points were counted in each patient to validate the diagnosis.

Two patients received no medication at all. One patient received trimipramine (half-value period 23 h), one patient received dulexetine (half-value period 8–17 h), two patients received pregabaline (half-value period 6 h), two patients received tilidine (half-value period 3–5 h) and amitryptiline (half-value period 8–51 h), one patient received fluoxetine (half-value period 4–6 h, tolperisone 2.5 h and promethazine 7 h). Absence of analgesic and anti-depressant medication at least for 24 h prior to the measurement was required. Although we cannot rule out the possibility that medication has not been washed out entirely, a longer period of medication absence was not tolerated by the patients and thus, was not possible. Since the half-value period of amitryptiline only exceeded the current wash-out period of at least 24 h, we would rule out an effect of medication on significant differences between FMS patients and control subjects.

Additionally, 10 subjects with masochistic behaviour (38.8±3.7 years; 5 male) participated in the present study which were acquired via internet boards. Again, two subjects were acquired through cooperation with the Institute of Neuropsychology and Clinical Psychology, Central Institute of Mental Health, Mannheim, Germany. MB was assessed according to DSM-IV-TR criteria [21]. In all subjects MB was practiced within a sexual context approximately once a week. Subjects with prevailing of sadistic behaviour as well as subjects with acute or chronic psychiatric disorders were excluded from the study. Healthy subjects matched with respect to age, gender and handedness served as control subjects for both groups, respectively (MB controls; 40.5±3.8 years; FMS controls: 53.9±3.2 years). All participants received financial compensation. Data of one FMS patient and one MB subject were not analyzed due to insufficient data quality. Consequently, data of the corresponding control subjects were excluded from the analysis.

A conditioning test stimulation paradigm was used while neuromagnetic signals were recorded. To this end, non-painful electrical pulses activating the tactile afferents of the superficial branch of the radial nerve of the right hand were applied as test stimuli (TS) 500 ms after a conditioning stimulus (CS). CS were either electrical stimuli at the same location and intensity as the TS or a slightly painful nociceptive cutaneous laser stimulation applied to the dorsum of the right hand. Each pair was separated by intervals varying between 4 and 6 seconds. For each condition (laser, tactile) 120 pairs were administered to each participant.

Tactile stimuli were electrical pulses of 0.3 ms duration and constant square-wave currents. Electrical pulses were delivered using a Grass S 88 stimulator (Grass Medical, Quincy, Mass., USA) and adhesive electrodes which were fixed over the supply area of the right radial nerve (i.e. over the wrist on the thumb side). Electrodes had a diameter of 0.5 cm. Subjects reported discernible, brief, non-painful touch-like stimuli during stimulation. Laser stimuli were applied using a YAG-laser (Carl Baasel Lasertechnik, Starnberg, Germany) with a wavelength of 2,000 nm, pulse duration of 1 ms and a spot diameter of 6 mm. Stimulus timing was realized using E-prime (Psychology Software Tools Inc.).

### Questionnaires

The German version of the State-Trait Anxiety Inventory (STAI) [22], the Beck's Depression Inventory (BDI) [23] and the Pain Catastrophizing Scale (PCS) [24] were administered to all participants immediately before MEG recordings. STAI determines positive and negative descriptions of oneself ranging between 1 (not applicable at all) and 4 (highly applicable). Values were determined for state anxiety (STAI-S) and trait anxiety (STAI-T), separately. BDI ranges between 0 (unsuggestive of depression) and 3 (suggestive of depression). PCS ranges between 0 (no negative pain related thoughts during painful situations) and 4 (permanent negative pain related thoughts).

Ratings of each questionnaire were summed for each individual and finally the mean sum score was calculated across participants for STAI-S, STAI-T, BDI and PCS, separately. Sum scores range between 20 and 80 (STAI-T and STAI-S, respectively), between 0 and 63 (BDI) and between 0 and 52 (PCS).

### Psychophysics

Individual detection thresholds for tactile and laser stimulation, respectively, as well as laser induced pain thresholds were determined by the method of limits of repeated ascending and descending series. Tactile stimulus intensities were set to the twofold of the individual sensory detection threshold.

After each laser stimulus the laser was moved a few millimetres in order to avoid tissue injury. According to Bromm et al. [25], subjects characterized the quality of sensations following laser stimulation verbally (i.e. touch, warm, tingling, pricking, burning). Pain thresholds were determined at intensities yielding pinprick sensations. Stimulus intensity was set to the twofold of the individual pain ratings which were attained between 3 and 5 in each subject.

Additionally, subjects rated pain intensity and associated feelings of pleasantness vs. unpleasantness by means of a visual scale immediately after the measurement. Pain ratings were determined by a numerical rating scale ranging from 0 (no pain) to 10 (worst possible pain). Ratings of pleasantness vs. unpleasantness ranged between -10 indicating that pain stimuli were extremely unpleasant and 10 suggesting that pain stimulation was highly pleasant. Finally, subjects were asked to evaluate the intensity of the CS and the TS across each measurement to estimate whether the subjective stimulation intensity differed between conditions (i.e. facilitation and attenuation).

### MEG data

MEG data were measured using a helmet-shaped 122-channel whole-head MEG-System (Neuromag™). During data acquisition subjects were comfortably seated in a magnetically shielded room. Both arms rested on wooden panels fixed laterally to the chair. MEG signals were recorded with a band-pass filter of 0.03–330 Hz, digitized with 1,000 Hz, and stored digitally for off-line analysis. Eye blinks were controlled by vertical electrooculogram (EOG).

High-resolution T1-weighted magnetic resonance images (MRI) were obtained from each subject. Co-registration between MRI and MEG data was achieved by localizing three anatomical landmarks (nasion, left and right preauricular points) in each individual and measuring the magnetic signals of four coils placed on the scalp.

Brain signals were averaged from -300 to 300 ms with respect to TS onset. In order to obtain information about processing of tactile stimuli without preceding CS, brain signals were additionally averaged time-locked to the tactile CS from -300 to 300 ms. The first five seconds of each run were omitted from averaging. Evoked responses were analyzed from 100 ms prior to 300 ms after stimulus onset. Somatosensory evoked fields (SEFs) were analyzed by means of a spatio-temporal source model. The location, orientation and amplitude of the best fitted equivalent current dipoles were estimated within a boundary element volume conductor model. Only sources accounting for more than 85% of the field variance were accepted. Sources were superposed on the individual MRI scans to delineate the anatomical localization. Individual sources were spatially normalized to Talairach space.

The time course of each source was determined by keeping the location and orientation fixed, while activation strengths were allowed to vary over time. Mean amplitude peaks were determined for each individual source and averaged across subjects. Paired comparisons between FMS patients and their respective control group and MB subjects and their control group were calculated using Mann-Whitney-U-Test for independent samples. Comparison between conditions within groups was calculated with Friedman tests for multiple related samples and post-hoc comparisons. For correlation analysis we used Spearman rank order correlation. All statistics were calculated two-tailed. P-values were corrected for multiple testing. Since no differences between both control groups were found, data were pooled for illustration. Nevertheless, all statistical comparisons were performed between FMS patients and controls and between MB subjects and the respective control group, separately.

## Results

### Clinical pain ratings of FMS patients

Mean pain ratings prior to the MEG measurement were 4.7±06. During the last four weeks mean pain intensity was 6.3±0.7. Mean number of tender points was 13.2±1.0 and overall range was 11–18.

### Questionnaires

**Figure 1:**
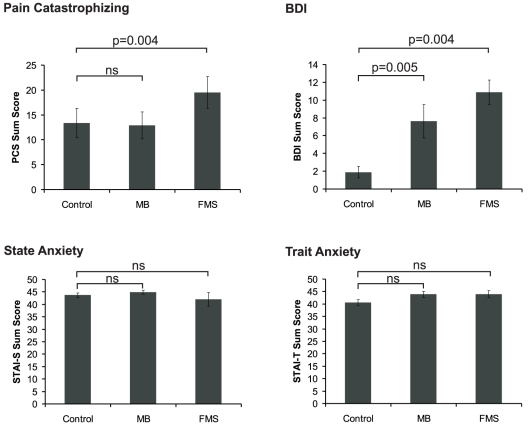
Pain catastrophizing (PCS), BDI, State Anxiety (STAI-S) and Trait Anxiety (STAI-T) sum scores in control subjects, subjects with masochistic behaviour (MB) and fibromyalgia patients (FMS). Error bars indicate standard error of mean (s.e.m.). Please note that statistics have been calculated for FMS and MB and their respective control subjects, separately. For simplification data from both control groups were pooled.

Pain catastrophizing was significantly increased in FMS patients as compared to control subjects (U = 7.00, p = 0.004). MB subjects as well as FMS patients had significantly higher BDI scores than control subjects (MB: U = 6.5, p = 0.005; FMS: U = 7.5, p = 0.004). Nevertheless, values were below 11 indicating that none of the subjects suffered from depression. No significant differences of trait or state anxiety between groups were found. Results are summarized in figure 1.

### Psychophysics

**Figure 2:**
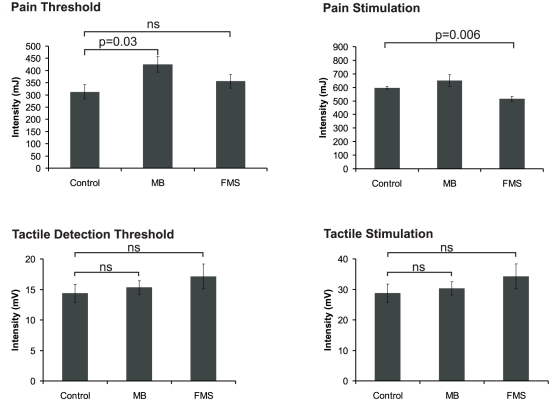
Summary of psychophysics in control subjects, subjects with masochistic behaviour (MB) and fibromyalgia patients (FMS). Shown are mean thresholds for pain and tactile stimuli (left) and intensities (right) for laser and tactile stimulation.

Neither tactile nor laser detection thresholds differed between MB subjects and FMS patients and the respective control group (p>0.500). Pain thresholds did not differ between FMS patients and control subjects (U = 23.5, p = 0.236) but were significantly higher in MB than in control subjects (U = 11.5, p = 0.028). Intensity of laser stimulation was significantly lower in FMS patients (U = 8.0, p = 0.006) but did not differ between MB subjects and controls (U = 18.0, p = 0.093). Results are summarized in figure 2.

Laser stimulation yielded mean pain ratings of 4.3±0.9 in controls, 3.1±0.6 in MB subjects and 4.3±0.4 in FMS patients. Statistical analysis revealed no significant differences between groups (p>0.10). Pleasantness vs. unpleasantness ratings revealed -1.5±0.7 in control subjects, 4.5±1.2 in MB subjects and −4.0±1.3 in FMS patients. Statistical analysis revealed higher positive ratings in MB as compared to the appendant control group (U = 4.0, p = 0.006) while ratings of FMS patients and controls did not differ significantly (U = 16.5, p = 0.3). Tactile stimulation was evaluated as discernible but non-painful by all subjects. Additionally, subjects reported no differences of subjective stimulation intensities between conditions.

### MEG recordings

Analysis of MEG data with respect to tactile CS revealed a well-known SEF sequence [26] with peaks at 39.7±1.8, 75.4±6.8, 117.7±4.6 and 120.3±6.5 ms in control subjects. No significant latency difference between MB, FMS patients and the respective control groups occurred (p>0.1). Analysis of MEG data with respect to tactile TS revealed no significant latency differences between conditions and groups (p>0.1).

**Figure 3:**
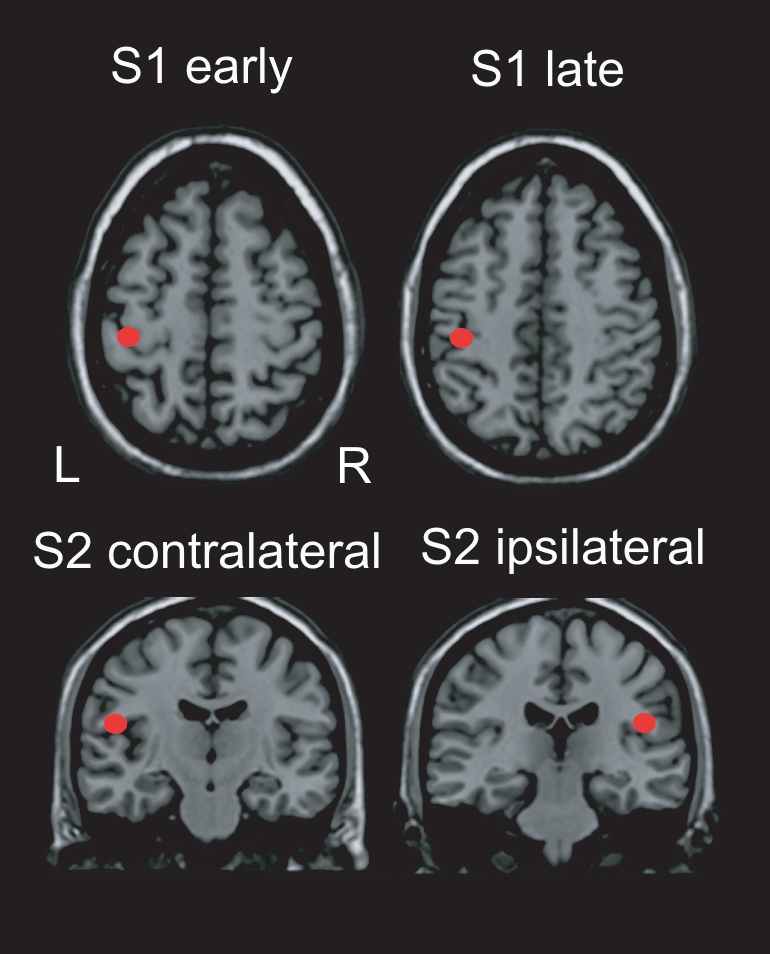
Mean localization of the early S1, late S1 and bilateral S2 responses superposed on the Talairach brain.

In all subjects, responses to stimulation were sufficiently explained by a four dipole model fitted at magnetic global field power [26]. Mean Talairach coordinates were −37.2 −27.1 46.7 mm (S1 early), −38.6 −27.3 43.3 mm (S1 late), −43.9 −24.7 5.8 mm (S2 contralateral), 46.2 −12.0 6.2 mm (S2 ipsilateral). No significant localization difference between MB and controls and FMS and the corresponding control group was found (p>0.5). Mean source localizations in the Talairach space are depicted in figure 3.

**Figure 4:**
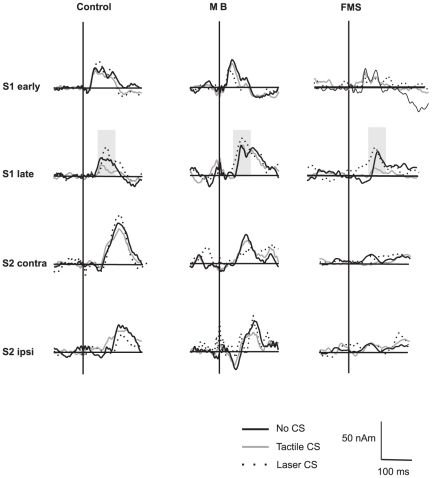
Mean source waveforms of early S1, late S1, and bilateral S2 averaged across control subjects, subjects with masochistic behaviour (MB) and fibromyalgia patients (FMS) depending on the modality of the preceding conditioning stimulus (e.g. no CS, tactile CS, laser CS). The grey rectangle depicts waveforms and time periods in which somatosensory processing varied depending on the preceding CS.

**Figure 5:**
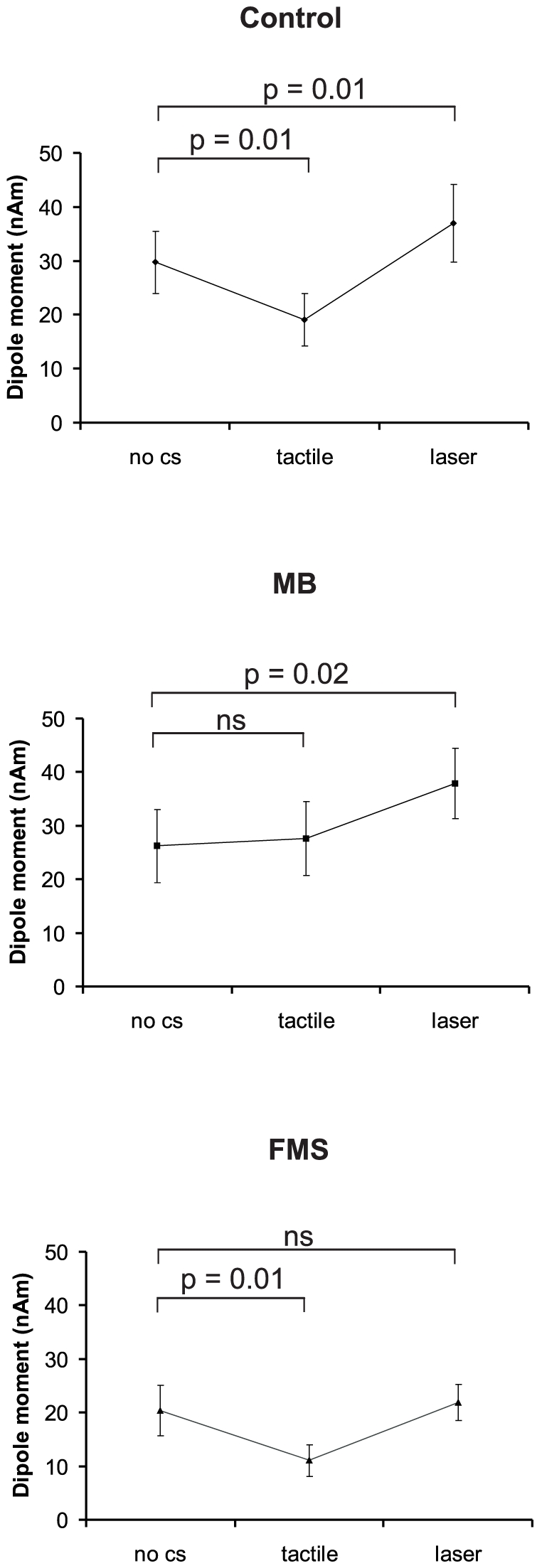
Mean dipole moment of the late S1 source depending on the modality of the preceding CS. Error bars indicate standard error of mean (s.e.m.).

In control subjects the late S1 amplitude varied depending on the modality of the CS (painful vs. tactile): A preceding tactile stimulus resulted in a significant decrease from 29.75±5.8 nAm (no CS) to 19.0±4.9 nAm (p = 0.01) indicating attenuation. Additionally, a significant increase following a preceding laser stimulus to 36.9±7.2 nAm (p = 0.01) was found suggesting facilitation. The preceding CS did not affect processing of TS in early S1 or bilateral S2 (p>0.3). In MB the late S1 amplitude was significantly increased following laser stimulation from 26.21±6.7 nAm to 37.9±6.5 nAm (p = 0.02). No significant difference following tactile stimulation was found (27.6±6.8 nAm; p = 0.60). Again, no significant effect in other sources occurred. In FMS patients the late S1 amplitude significantly decreased following tactile stimulation from 20.3±4.6 nAm to 11.1±2.9 nAm (p = 0.01) whereas a preceding laser stimulation did not result in significant changes (21.8±3.3 nAm; p = 0.63). Results are summarized in figures 4 and 5.

**Figure 6:**
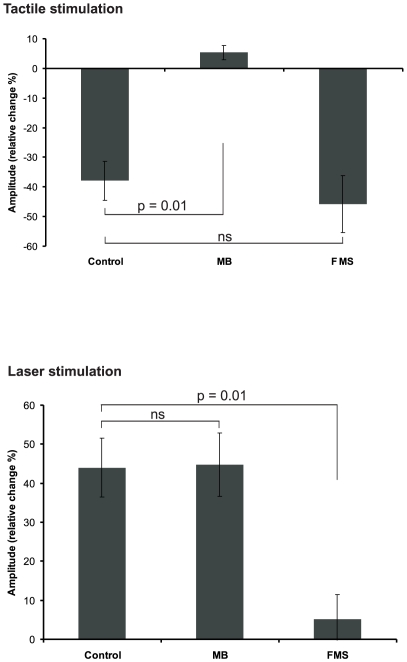
Relative amplitude changes of the late S1 source. Amplitudes of the no CS condition were set to 100%. Relative amplitudes of TS were calculated individually and averaged across subjects.

For the analysis between groups relative amplitude changes were calculated. To this end, amplitudes of the No-CS condition were set to 100%. In control subjects the late S1 amplitude following laser stimulation increased by 44.6%, whereas a decrease by 36.6% occurred following tactile stimulation. In MB preceding laser stimuli yielded an amplitude increase of 44.7% while it was increased by 5.3% following tactile CS. Comparison between groups revealed a significant difference of the tactile condition only (U = 5.0, p = 0.01). Conversely, in FMS patients tactile CS revealed a comparable decrease by 44.7% as in control subjects. Laser stimulation elicited an increase of 5.2% which was significantly smaller than in the control group (U = 3.0, p = 0.01). Results are summarized in figure 6. No significant differences of early S1 or S2 responses were found between groups.

In order to assess whether somatosensory excitability is affected by catastrophizing, depression, anxiety, stimulation intensity, pain rating or feelings of pleasantness vs. unpleasantness these measures were correlated with source amplitudes for each group. The analysis revealed a significant inverse correlation between late S1 amplitude and PCS in FMS patients only (Rho = −0.747, p = 0.033). Partial correlation analysis controlling for pain rating, pain evaluation (i.e. pleasant vs. unpleasant) and stimulation intensity revealed comparable results (controlling for pain rating: Rho = −0.731; controlling for pain evaluation: Rho = −0.799; controlling for stimulation intensity: Rho = −0.771).

## Discussion

The present data suggest altered modulation of somatosensory processing in subjects with masochistic behaviour and in patients with fibromyalgia syndrome. Alterations applied to the late but not to the early S1 response. Noteworthy, patients with FMS and subjects with masochistic behaviour showed reversed patterns of alterations. While in FMS patients a preceding tactile stimulus yielded decrease of the late S1 response following brief tactile stimulation, an increase of this amplitude following painful laser TS was not evident. Conversely, subjects with masochistic behaviour showed no amplitude decrease following tactile CS but the same amount of amplitude increase following laser CS as control subjects. Pain catastrophizing was inversely correlated with the amplitude of the late S1 source in FMS patients suggesting that reactivity of this source is modulated by specific pain related attitudes.

### Psychophysics

The present data suggest no differences of tactile as well as pain thresholds between FMS patients and control subjects. In MB increased pain thresholds as compared to control subjects were found. The first result is in line with previous findings indicating differences between FMS patients and control subjects to intense [18], [27] but not to weak stimulation [28]. These data led to the hypothesis that in FMS an inhibitory system which prevents healthy subjects from overstimulation might be deficient. Pain ratings revealed no differences between groups suggesting that although stimulation intensities were reduced in FMS, a comparable subjective pain sensation was elicited as in healthy control subjects. Nevertheless, it comes as a surprise that pain thresholds did not differ between FMS patients and controls. Currently, we can only speculate about possible causes but, our data imply that FMS patients were able to tolerate single painful stimuli well while repeated stimulation (i.e. during the experiment) were less tolerated resulting in reduced stimulation intensities. These data reveal a piece of evidence that pain thresholds are indeed altered in FMS patients. Alternatively, this result might be due to a restrictive aspect of the present data: the short wash-out period of 24 h. As mentioned above the half-value period of amitryptiline persists up to 51 h. Thus, we cannot completely rule out the possibility that the effects observed might be influenced by medication.

On a pleasantness vs. unpleasantness scale, MB subjects evaluated laser stimuli consistently as more pleasant than control subjects indicating that painful stimulation is evaluated as positive even in a setting in which masochistic behaviour is usually not practiced (i.e outside a sexual context).

### MEG data

Previous EEG studies suggest increased somatosensory evoked responses following painful stimulation in FMS patients using CO2 laser [29] or electrical stimulation [30] suggesting enhanced sensory processing in FMS. In healthy subjects, repetition of tactile stimuli yields reduced EEG and MEG responses - a well-known psychophysiological phenomenon called *sensory gating* - which might reflect the capability of the brain to filter irrelevant information. The present results revealed normal *sensory gating* in FMS but a lack of such attenuation in subjects with masochistic behaviour. Thus, one might argue that FMS patients principally have the capability to distinguish between relevant and irrelevant information in order to ignore innocuous stimuli. However, it should be stressed that the present results are at odds with previous data indicating a loss of attenuation following repeatedly presented painful [15] and non-painful tactile stimuli [14] in FMS by means of EEG. Although not entirely clear, one might speculate that medication or differences of pain related attitudes might have yielded this discrepancy.

Interestingly, the present results suggest that *sensory gating* seems to be altered in MB subjects in that sense that innocuous stimuli are not dealt as irrelevant. Although speculative, one might argue that this result reflects a kind of floor effect possibly due to reduced cortical excitability. More precisely, in these subjects excitability might be reduced so that additional suppression is not possible. Thus, stimuli yielding increased activity in control subjects like pain might result in “normal” activity in masochistic subjects. This might explain why these subjects do not perceive painful stimuli as aversive but as normal.

Preceding painful stimuli have been shown to facilitate tactile processing as indicated by increased evoked responses using MEG [7]. It has been argued that this facilitative effect might represent a neurophysiological correlate of the alerting function of pain. Along this line, previous studies suggest that attention indeed affects central pain processing (e.g. [31], [32], [33]). In the present data a preceding painful laser stimulus yielded facilitation in subjects with masochistic behaviour but not in FMS patients. This implies that FMS patients are less likely to draw their attention to painful stimuli – possibly in order to avoid pain perception. We realize that this interpretation is highly speculative at the moment since attention was not controlled in the present study. As an alternative interpretation it has been argued that in FMS patients an inhibitory system might be deficient. The present data imply increased inhibition resulting in a lack of facilitation following painful CS. Both lines of interpretation are not mutually exclusive since the inhibitory system might be driven by attention. Along this line, in healthy subjects painful stimuli might alert the somatosensory system and as a result inhibition is reduced. In contrast, in FMS painful stimulation results in increased inhibition possibly mediated by reduced attention to painful CS.

As a further interpretation one might argue that the subjective TS intensity might differ between conditions. But, none of the subjects reported such differences ruling out this hypothesis.

FMS patients had significantly increased PCS scores as compared to control subjects - a well known symptom in FMS (for review see [34]). It has been argued that catastrophizing affects central pain processing [35]. Accordingly, in FMS patients an inverse relation between PCS and late S1 amplitude was found. More precisely, the stronger pain related catastrophizing was the more distinct attenuation was. This result supports the hypothesis that the late S1 component is modulated by pain related attitudes.

All in all, the present results imply altered cortical reactivity of the primary somatosensory cortex in FMS patients and subjects with masochistic behaviour suggesting that individual pain experience affects tactile processing in S1.
